# Chest CT Scan Features to Predict COVID-19 Patients' Outcome and Survival

**DOI:** 10.1155/2022/4732988

**Published:** 2022-02-26

**Authors:** Mohammad-Mehdi Mehrabi Nejad, Aminreza Abkhoo, Faeze Salahshour, Mohammadreza Salehi, Masoumeh Gity, Hamidreza Komaki, Shahriar Kolahi

**Affiliations:** ^1^Department of Radiology, School of Medicine, Advanced Diagnostic and Interventional Radiology Research Center (ADIR), Imam Khomeini Hospital, Tehran University of Medical Sciences, Tehran, Iran; ^2^Department of Infectious Diseases and Tropical Medicines, Tehran University of Medical Sciences, Tehran, Iran; ^3^Brain Engineering Research Center, Institute for Research in Fundamental Sciences (IPM), Tehran, Iran

## Abstract

**Background:**

Providing efficient care for infectious coronavirus disease 2019 (COVID-19) patients requires an accurate and accessible tool to medically optimize medical resource allocation to high-risk patients.

**Purpose:**

To assess the predictive value of on-admission chest CT characteristics to estimate COVID-19 patients' outcome and survival time.

**Materials and Methods:**

Using a case-control design, we included all laboratory-confirmed COVID-19 patients who were deceased, from June to September 2020, in a tertiary-referral-collegiate hospital and had on-admission chest CT as the case group. The patients who did not die and were equivalent in terms of demographics and other clinical features to cases were considered as the control (survivors) group. The equivalency evaluation was performed by a fellowship-trained radiologist and an expert radiologist. Pulmonary involvement (PI) was scored (0–25) using a semiquantitative scoring tool. The PI density index was calculated by dividing the total PI score by the number of involved lung lobes. All imaging parameters were compared between case and control group members. Survival time was recorded for the case group. All demographic, clinical, and imaging variables were included in the survival analyses.

**Results:**

After evaluating 384 cases, a total of 186 patients (93 in each group) were admitted to the studied setting, consisting of 126 (67.7%) male patients with a mean age of 60.4 ± 13.6 years. The PI score and PI density index in the case vs. the control group were on average 8.9 ± 4.5 vs. 10.7 ± 4.4 (*p* value: 0.001) and 2.0 ± 0.7 vs. 2.6 ± 0.8 (*p* value: 0.01), respectively. Axial distribution (*p* value: 0.01), cardiomegaly (*p* value: 0.005), pleural effusion (*p* value: 0.001), and pericardial effusion (*p* value: 0.04) were mostly observed in deceased patients. Our survival analyses demonstrated that PI score ≥ 10 (*p* value: 0.02) and PI density index ≥ 2.2 (*p* value: 0.03) were significantly associated with a lower survival rate.

**Conclusion:**

On-admission chest CT features, particularly PI score and PI density index, are potential great tools to predict the patient's clinical outcome.

## 1. Introduction

Coronavirus disease 2019 (COVID-19), caused by severe acute respiratory syndrome coronavirus-2 (SARS-CoV-2), was officially announced as a pandemic by the World Health Organization on March 11, 2020 [1]. Even though most of the patients experience mild symptoms, some may develop a severe type of disease and it may progress to acute respiratory distress syndrome (ARDS) [2]. The global death-to-case ratio is estimated to be 3.5% [3]. However, it varies geographically probably due to the local preventive measures and medical resources. Therefore, we need to improve the admitted patients' initial triage not only to optimize the allocation of the medical resources to high-risk patients and minimize the mortality rate accordingly but also to more accurately and reliably predict the outcome of the patients.

Chest computed tomography (CT) scan, as the conventional and relatively accessible imaging modality for pneumonia diagnosis and follow-up, is confirmed to have high diagnostic and prognostic values in the current outbreak of COVID-19 [4, 5]. Chest CT findings mainly consist of ground-glass opacities (GGOs), multifocal patchy consolidation, and interstitial changes with a peripheral distribution [6–8]. Efforts have been made to underpin the predictive factors for mortality [9]. Among all, the main factors consist of age, underlying disease (i.e., immunocompromised patients and preexisting cardiovascular and pulmonary disorders), laboratory findings (i.e., D-dimer level, neutrophil-to-lymphocyte ratio (NLR), and lymphocyte count), and most recently, imaging features [10–13]. However, there is still no consensus on the factor with the highest predictive value. Besides, almost all previous studies were retrospective cross-sectional and nonsurvivors comprised only a small population [14, 15], which further limit the application of the findings.

In this regard, we aimed to utilize the vastly used modality-chest CT scan, and we hypothesize that on-admission chest CT findings could serve as a potential deterministic factor for risk stratification in hospitalized COVID-19 patients. In order to evaluate the proposed notion, we conducted a case-control study and assessed the adjusted predictive value of CT scan in terms of mortality rate for admitted COVID-19 patients.

## 2. Materials and Methods

### 2.1. Study Design and Participants

**Figure 1:**
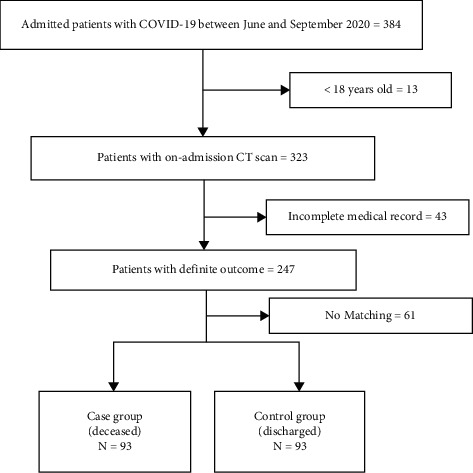
Flow diagram of the study.

This case-control study was reviewed and approved by the Institutional Review Board of our university. Given the retrospective design of the study and anonymous use of medical records, informed consent requirement was waived by the ethics committee of our institute (IR.TUMS.VCR.REC.1399.054). The current case-control study was carried out in a referral tertiary university hospital from June to September 2020. This study evaluated individuals with the following conditions: (a) all hospitalized COVID-19 patients in whom COVID-19 diagnosis was confirmed by positive real-time reverse transcription-polymerase chain reaction (rRT-PCR) assay on nasopharyngeal or oropharyngeal swap or endotracheal aspirate samples; (b) age equal or greater than 18 years; and (c) a definite outcome of either death or hospital discharge. Case patients consisted of patients who met the inclusion criteria and were deceased. Control patients included discharged patients with equivalent demographic (age and sex) and clinical (underlying diseases and laboratory findings) features. The equivalency evaluation was performed by a fellowship-trained radiologist and an expert epidemiologist. The flow diagram of the study is presented in Figure 1. Of note, admission, discharge criteria, and treatment of all patients were based on the national protocol of COVID-19.

### 2.2. Data Collection

#### 2.2.1. Population Characteristics

The recorded attributes of patients consisted of the following: (a) *demographic characteristics*, age and sex; (b) *on-admission vital signs*, temperature (T-Celsius), oxygen saturation (SpO_2_), heart rate (HR-per minute), respiratory rate (RR-per minute), and blood pressure (BP-mmHg); (c) *survival time*, days from admission to death in nonsurvived patients; (d) *underlying diseases*, hypertension (HTN), diabetes (DM), respiratory disease (asthma, COPD, ILD, or bronchiectasis), malignancies (solid or hematological malignancies), immunocompromised conditions (chemoradiation therapy and long-term corticosteroid usage), and hypothyroidism; and (f) *laboratory findings*, white blood cell including neutrophil and lymphocyte counts, hemoglobin, platelet, creatinine, urea, international normalized ratio (INR), partial thromboplastin time (PTT), D-dimer, lactate dehydrogenase (LDH),C-reactive protein (CRP), and pro-b-type natriuretic peptide (Pro-BNP).

#### 2.2.2. Image Acquisition

All chest CT images were acquired at the time of admission, in the supine position, with full inspiration with no contrast injection. Examinations were performed on either the Siemens Somatom Emotion (16 slices, Erlangen, Germany) or the Lightspeed 64-detector CT (GE Healthcare, Milwaukee, USA) MDCT scanner. The imaging parameters were set at 5–6 mm section thickness, beam collimation of 0.6–2 mm, 120 kVp tube voltage, tube current of 150–250 mAs, tube rotation speed of 0.75 seconds, and gantry rotation time of 0.5–0.75 s, reconstructed with a mediastinum B20f smooth kernel and a lung B70f sharp kernel (Siemens Healthineers, Erlangen, Germany); coronal and sagittal multiplanar reconstructions were also available with a reconstructed slice thickness of 1.2 mm.

#### 2.2.3. Image Interpretation

Two fellowship-trained diagnostic imaging radiologists, with respective 9 and 13 years of experience in thoracic radiology and blinded to patients' outcomes, independently interpreted chest CT images' findings. All CT images were reviewed on both lung- and mediastinal-window settings. The intraclass correlation coefficient (ICC) was calculated to assess inter-rater reliability. If ICC was less than 0.8, any disagreement in image interpretation for the case was discussed until resolved. If ICC was greater than or equal to 0.8, the value reported by the radiologist with higher experience was recorded.

**Figure 2:**
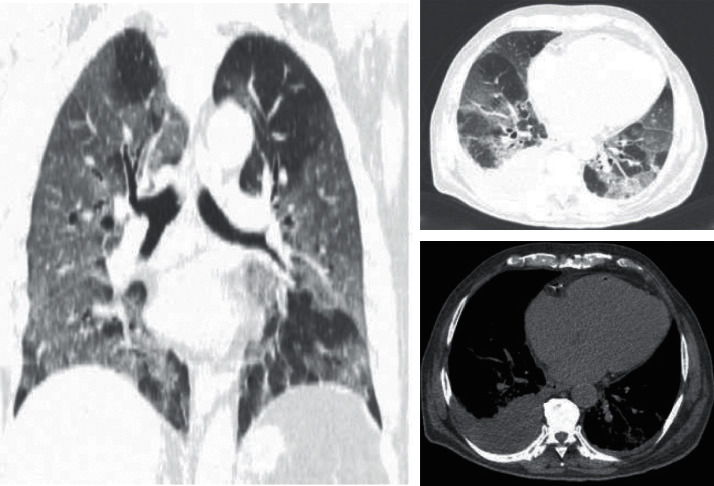
A 76-year-old male patient with diabetes deceased due to COVID-19. Chest CT scan ((a) coronal view, (b) axial view-lung window, and (c) axial view-mediastinal window) showed predominancy of ground-glass opacity (GGO) with the bilateral and diffuse distribution. Pulmonary involvement (PI) and PI density scores were 16 and 3.2, respectively. Additional findings included cardiomegaly and bilateral pleural effusion. He was considered a high-risk patient.

**Figure 3:**
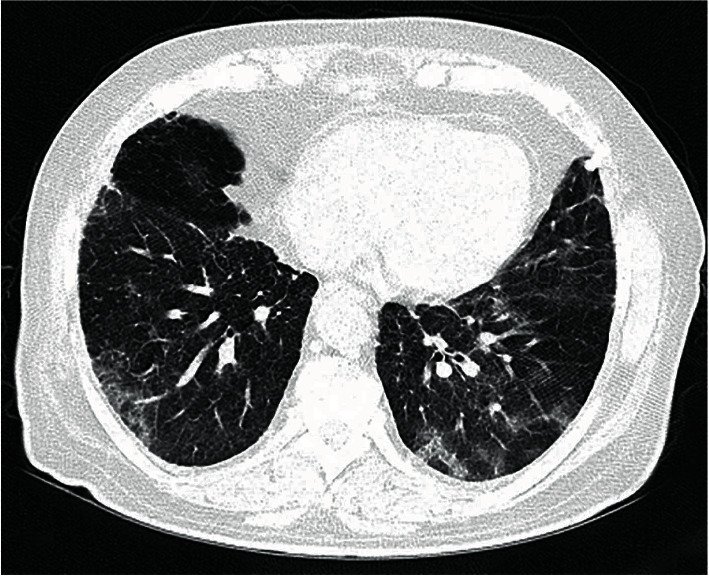
A 61-year-old female patient with hypertension and diabetes. Pulmonary involvement: predominancy of GGO with peripheral, pleural-based distribution. Total pulmonary involvement (PI) score and PI density index were 6 and 1.2, respectively, and she was stratified as a low-risk patient in death predictive models.

Chest CT scan findings were recorded according to the Fleischner Society glossary and published literature on viral pneumonia [16]. Chest CT scan features included the following: (a) *predominant pattern*, ground-glass opacification/opacity (GGO) (Figure 2), consolidation (Figure 3), and mixed; (b) *dominant distribution pattern*, peripheral (peripheral one-third of the lung) (Figure 3), axial (medial two-thirds of the lung), and diffuse (Figure 2); (c) *number of involved lobes*; (d) *other morphologies*, parenchymal band, crazy paving, reverse halo sign, or intralesional traction bronchiectasis; and (e) *additional findings*, cardiomegaly (Figure 2), pleural effusion (unilateral or bilateral) (Figure 2), pericardial effusion, emphysema, dilated pulmonary trunk, pleural thickening, and mosaic attenuation.

#### 2.2.4. Pulmonary Involvement (PI) Scoring System

To assess PI, a semiquantitative scoring tool was proposed and used [17]. All five lung lobes (right upper lobe (RUL), right middle lobe (RML), right lower lobe (RLL), left upper lobe (LUL), and left lower lobe (LLL)) were visually reviewed for GGO and consolidation. Then, a score from 0 to 5 was assigned to each lobe according to involvement percentage (0: no involvement; 1: ≤5%; 2: 6–25%; 3: 26–50%, 4: 51–75%; and 5: ≥76%). The total PI score was calculated as the sum of all five lobes' scores. The PI score ranged from 0 (no involvement) to 25 (maximum involvement). Finally, the PI density index was calculated by dividing the total PI score by the number of involved lobes.

### 2.3. Statistical Analysis

We performed the analyses in SPSS for Windows ver. 18 (Chicago, IL, USA). Descriptive data are presented as mean with standard deviations (SD) for continuous variables and as frequency and percentage of the population for categorical variables. In order to evaluate whether the recorded data have a normal distribution, we used the Kolmogorov–Smirnov test. We conducted the comparisons by (a) the independent two-tailed sample *t*-test for continuous variables with normal distribution and the relevant degree of freedom; (b) the Mann–Whitney *U* test for the continuous variables with significant lack of normality variables; and (c) the Chi-square test for nominal variables. All *p* values less than 0.05 were considered statistically significant.

We used multivariate logistic regression to predict mortality probability with all imaging parameters as the independent variables. As previously stated, the statistically significant threshold was considered as a *p* value less than 0.05. To define optimum cutoff values for PI score and PI density index in outcome prediction, receiver-operating characteristic (ROC) curves were drawn and Youden's *J* index [18] was calculated. The area under the ROC curve (AUC) was considered as the indicator for ROC analysis efficacy.

To determine the impact of any independent variable on survival time, we implemented univariate Cox regressions (in nonsurvivors) considering all demographic, clinical, and imaging parameters as covariates in the model. Multivariate backward Cox regression was performed to find the final model in terms of which variables to include. Kaplan–Meier survival analysis was performed to calculate survival, and the log-rank test was used to compare the survival distribution of two subgroups of interest.

## 3. Results

### 3.1. Study Population Characteristics

After evaluating 384 cases, a total of 186 patients (93 in each group) were admitted to the studied setting, consisting of 126 (67.7%) male patients with a mean age of 60.4 ± 13.6 years (Figure 1). The most common underlying diseases were HTN (38.7%) and DM (34.9%) (Table 1).

Two groups were almost equivalent for demographic (age and sex) and clinical (underlying diseases and laboratory findings) variables. Table 1 further illustrates the homogeneity of cases and controls in the aforementioned characteristics. All ICCs for inter-rater reliability were >0.8 for all imaging parameters.

### 3.2. Chest CT Scan Findings

The most common CT features among survivors and nonsurvivors were GGO (65.6% and 68.8%), multilobar (95.7% and 98.9%), bilateral lobe involvement (93.5% and 94.6%), and lower lobe (RLL and/or LLL) involvement (94.6% and 98.9%), respectively. Predominant distribution patterns among the two groups were peripheral (47.3% in survivors) and axial (57.0% in nonsurvivors).

The mean PI score and PI density index in survivors vs. nonsurvivors were 8.9 ± 4.5 vs. 10.7 ± 4.4 (*p* value: 0.001) and 2.0 ± 0.7 vs. 2.6 ± 0.8 (*p* value: 0.01), respectively. In addition, nonsurvived patients had a higher involvement score for all single lung lobes (except RLL) and axial distribution (37.6% vs. 57.0%, *p* value: 0.01). However, the number of involved lobes, predominant pattern, and bilateral and lower lobe involvement did not show any significant difference in comparison (*p* values: 0.49, 0.63, 0.76, and 0.09) (Table 2).

Of additional findings and other morphologies, cardiomegaly (35.5% vs. 55.9%, *p* value: 0.005) (Figure 2), pleural effusion (9.7%% vs. 28.0%, *p* value: 0.001) (Figure 2), pericardial effusion (3.2% vs. 10.8%, *p* value: 0.04), dilated pulmonary trunk (5.4% vs. 19.4%, *p* value: 0.004), and pleural thickening (3.2% vs. 33.3%, *p* value:<0.001) were significantly more prevalent in the nonsurvived group. Table 2 demonstrates the details of patients' characteristics associated with death.

We exploited backward multivariate logistic regressions that included death as the outcome of interest and all imaging parameters with significant association in Table 2 as the independent variables. PI score (Ex(B): 1.10 (95% CI: 1.03–1.17), *p* value: 0.006)) and PI density index (Ex(B): 1.64 (95% CI: 1.10–2.43), *p* value: 0.02)) independently remained as significant variables in the models. The PI score with a cutoff value of 10 (AUC: 0.62 [0.54–0.70], Youden's J: 0.23, *p* value: 0.005) and PI density index with a cutoff value of 2.2 (AUC: 0.61 [0.52–0.69], Youden's J: 0.19, *p* value: 0.01) showed the best accuracy in predicting death (Figures 2–3).

### 3.3. Survival Analysis

**Figure 4:**
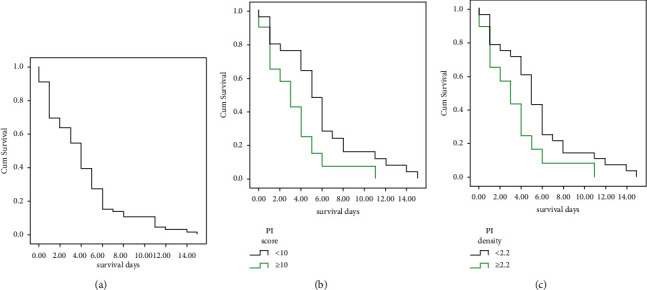
Kaplan–Meier survival curve. Estimated survival rate in (a) all deceased patients and comparisons based on (b) PI score and (c) PI density score.

The survival analyses were limited to nonsurvived patients. The mean survival was 4.1 ± 3.5 days, and the median survival was four days. The Kaplan–Meier survival function for death is illustrated in Figure 4. To determine the impact of independent variables on survival, we implemented univariate Cox regressions, considering demographic, clinical, and imaging variables as independent variables. Statistically significant variables included PI score ≥ 10 (*p* value: 0.02) and PI density ≥ 2.2(*p* value: 0.03). Afterward, we fitted a multivariate backward Cox regression on the variables with a significant univariate association: PI score ≥ 10 (*p* value: 0.03) remained significant at the final step (Table 3).

Further analyses using the Kaplan–Meier survival function and the log-rank test revealed that survival was significantly different in two subgroups according to PI score ≥ 10 (*χ*^2^ = 7.05, *p* value: 0.008) and PI density ≥ 2.2 (*χ*^2^ = 6.58, *p* value: 0.01) (Figure 4).

## 4. Discussion

There is still no consensus on the clinical and imaging factors that are associated with and affect COVID-19 patients' outcomes and survival. Our findings contribute to the existing literature by illustrating that patients with higher PI scores, PI density index, axial distribution, cardiomegaly, or pleural or pericardial effusion are more likely to decease. The PI density score is our novel suggested index to distinguish between patients with the same PI score but a different number of involved lobes because it showed a stronger mortality prediction power.

Previously reported chest CT features to predict COVID-19 patients' mortality mainly included crude lung involvement score, number of involved lobes, bilateral or lower lobe involvement, and diffuse pattern [14, 19–25]. In line with our findings, yet in contrast to previous reports, Yuan et al. [14] reported no significant association of lower lobe or bilateral lung involvement with death. Nevertheless, a significantly higher CT score was the most common reported feature to predict death [14, 19–23, 25]. Limited previous studies also reported cutoff values using totally different scoring systems [14, 21, 23]. For instance, Francone et al. [23] retrospectively evaluated 130 COVID-19 patients (20 patients deceased) and recommended the CT score ≥ 18 (out of 25) as the predictive factor for death. Another study used a 20-scale scoring system in 50 patients (23 deceased, 27 survived) and suggested a cutoff value of 12 with 0.79 AUC in predicting mortality [21]. Moreover, a study used a 72-scale CT score and reported 85% sensitivity and specificity in predicting the mortality of patients with a cutoff value of 24.5 [14]. In general, all previous studies were retrospective and nonsurvivors mostly comprised a small proportion of the studied population. Hence, differences in data sampling and study design (case-control vs. cross-sectional), as well as group matching, in our study remarkably improved the validity of results. Furthermore, details of chest CT findings were not fully reported in previous studies, and there is no consistency on the value of additional findings, including crazy paving and pleural effusion in predicting the outcome [19, 21, 25], which are both depicted in this study.

Further analyses on patients' survival demonstrated that PI score <10 and PI density index <2.2 were significantly associated with higher survival. To the best of our knowledge, imaging factors associated with the survival days of COVID-19 patients were addressed only in one retrospective study on 20 deceased patients [23]. In line with our findings, they found significantly lower survival days in patients with higher (≥18) vs. lower (<18) CT scores over a 24-day follow-up period [23].

Considering the limitations of the study, we still performed the largest case-control investigation on evaluating the clinical and imaging factors to predict COVID-19 patients' outcomes and survival. We provided a novel semiquantitative scale with a defined optimum cutoff, which could serve to better identify high-risk patients and recognize patients with a higher demand for critical care but similar clinical conditions compared to other patients. We posit that patients with a PI score ≥ 10 and a PI density score ≥ 2.2 should be carefully cared for and receive aggressive treatment, as they are highly susceptible to death. Hence, radiologists are expected to report PI and PI density scores in their everyday practice to help clinical physicians manage patients more effectively and efficiently and consequently minimize COVID-19-related mortality. Nevertheless, our findings must be interpreted in light of some limitations. Firstly, the study context is prone to a biased selection of cases and controls. To elaborate, the setting is a tertiary and referral hospital for COVID-19 patients, and the patients who get admitted are probably more clinically severe than the cases in the general population with similar paraclinical and demographic features. On the other hand, there are reports on factors, such as immunological factors and predispositions in patients, that might have an impact on the survival rate of the COVID-19 cases [26,27]. However, we did not have other available data to incorporate in the study. In addition, the admitted patients are not equivalent to outpatient cases in the predicted mortality rate. For example, outpatient cases might have an even lower mortality rate with a similar PI score because they are in a relatively better clinical condition. Another limitation is that we did not follow the discharged patients to evaluate whether they were deceased. The interval time between symptom onset and admission was not exactly the same in all patients; however, we used the on-admission CT scans to minimize this bias. Although all patients were treated based on a unique national protocol, minor treatment differences based on physician clinical judgment could affect mortality and survival, which were not assessed in the present study. This study was conducted before the national COVID-19 vaccination program and further investigations on a larger population after vaccination are recommended to confirm our findings [28].

In conclusion, patients with a higher PI score, PI density index, axial distribution, cardiomegaly, or pleural or pericardial effusion are more susceptible to poor prognosis. Survival analyses revealed that a PI score ≥ 10 and PI density score ≥ 2.2 were significantly associated with lower survival and those patients should be prioritized for higher medical attention. Initial chest CT examination may be able to help in predicting the patient's outcome and survival.

## Figures and Tables

**Table 1:** Details of demographic and clinical data of patients and differences between two groups.

Variables	All patients, N = 186	Survivors, N = 93	Nonsurvivors, N = 93	*P* value
Demographic data				
Age^*∗*^	60.4 (13.6)	58.7 (13.9)	62.2 (13.1)	0.08
Gender				
Male	126 (67.7)	61 (65.6)	65 (69.9)	0.53
Female	60 (32.3)	32 (34.4)	28 (30.1)	

Clinical data				
Vital signs				
RR^*∗*^	15.1 (9.1)	25.3 (5.1)	24.9 (11.8)	0.79
RR>24	102 (54.8)	51 (54.8)	51 (54.8)	1
Systolic BP^*∗*^	122.2 (20.1)	124.3 (18.0)	120.1 (21.8)	0.15
Diastolic BP^*∗*^	75.2 (12.7)	78.0 (10.9)	72.5 (13.7)	0.03
PR^*∗*^	96.9 (18.8)	97.4 (18.6)	96.4 (19.2)	0.70
Temperature^*∗*^	37.5 (0.8)	37.4 (0.8)	37.5 (0.9)	0.19

Hospitalization duration^*∗*^				
Total admission days	10.6 (8.9)	9.6 (8.0)	11.6 (9.7)	0.11
ICU days	5.2 (8.1)	3.0 (5.8)	7.4 (9.5)	<0.001

Underlying disease				
HTN	72 (38.7)	32 (34.4)	40 (43.0)	0.29
DM	65 (34.9)	36 (38.7)	29 (31.2)	0.35
Respiratory disease	14 (7.5)	9 (9.7)	5 (5.4)	0.40
Immunocompromised	18 (9.7)	7 (7.5)	11 (11.8)	0.45
Hypothyroidism	13 (7.0)	9 (9.7)	4 (4.3)	0.24

Laboratory findings^*∗*^				
WBC	8.9 (4.7)	8.3 (4.3)	9.3 (5.0)	0.28
Neutrophil	7.0 (3.9)	6.5 (3.9)	7.4 (3.8)	0.24
Lymphocyte	1.4 (3.9)	1.2 (0.7)	1.5 (2.8)	0.52
Hemoglobin	12.5 (2.7)	12.8 (2.4)	12.3 (2.9)	0.40
Platelet	209.3 (95.2)	219.2 (110.9)	202.2 (82.4)	0.39
Cr	1.7 (1.6)	1.7 (1.3)	1.7 (1.8)	0.99
Urea	58.3 (60.8)	49.8 (36.1)	64.5 (73.4)	0.24
INR	1.3 (0.8)	1.4 (1.1)	1.2 (0.5)	0.51
PTT	41.4 (22.2)	43.4 (25.1)	40.0 (19.8)	0.47
D-dimer	3364.1 (3210.0)	3450.7 (3344.9)	3147.7 (3316.3)	0.88
LDH	661.9 (287.3)	676.6 (269.7)	649.1 (305.7)	0.72
CRP	123.8 (73.0)	113.3 (72.1)	130.6 (73.4)	0.24
Pro-BNP	5329.9 (10289.0)	4505.8 (10687.2)	6085.2 (10324.3)	0.72

^*∗*^Mean (standard deviation); all other variables reported as N (%). RR = respiratory rate; BP = blood pressure; PR = pulse rate; HTN = hypertension; DM = diabetes; ICU = intensive care unit; WBC = white blood cell; Cr = creatinine; INR = international normalized ratio; PTT = partial thromboplastin time; LDH = lactate dehydrogenase; CR = C-reactive protein; Pro-BNP = pro-b-type natriuretic peptide.

**Table 2:** Radiologic findings in all patients and differences between the two groups.

Variables	All patients, N = 186	Survivors, N = 93	Nonsurvivors, N = 93	*P* value
PI scores				
RUL total score^*∗*^	2.0 (1.1)	1.8 (1.0)	2.2 (1.1)	0.02
RML total score^*∗*^	1.5 (1.0)	1.3 (0.9)	1.6 (1.0)	0.03
RLL total score^*∗*^	2.3 (1.1)	2.1 (1.1)	2.4 (1.1)	0.15
LUL total score^*∗*^	2.0 (1.1)	1.7 (1.1)	2.2 (1.1)	0.02
LLL total score^*∗*^	2.2 (1.2)	2.0 (1.2)	2.3 (1.2)	0.03
Total lung GGO score^*∗*^	6.7 (4.5)	6.2 (4.2)	7.2 (4.8)	0.13
Total lung consolidation score^*∗*^	3.1 (3.2)	2.6 (3.0)	3.6 (3.5)	0.05
Total PI score^*∗*^	9.8 (4.5)	8.9 (4.5)	10.7 (4.4)	0.001
PI density index^*∗*^	2.1 (0.8)	2.0 (0.7)	2.6 (0.8)	0.01

Predominant pattern				
GGO	125 (67.2)	61 (65.6)	64 (68.8)	0.63
Consolidation	61 (32.8)	32 (34.4)	29 (31.2)	

Dominant distribution of lesions				
Peripheral	70 (37.6)	44 (47.3)	26 (28.0)	0.01
Axial	88 (47.3)	35 (37.6)	53 (57.0)	
Diffuse	28 (15.1)	14 (15.1)	14 (15.1)	
No. of involved lobes	4.5 (1.0)	4.5 (1.1)	4.6 (0.9)	0.49

Monolobar				
1	5 (2.7)	4 (4.3)	1 (1.1)	0.05
Multilobar				
2	5 (2.7)	1 (1.1)	4 (4.3)	
3	12 (6.5)	3 (3.2)	9 (9.7)	
4	15 (8.1)	11 (11.8)	4 (4.3)	
5	149 (80.1)	74 (79.6)	75 (80.6)	

Laterality				
Unilateral	11 (6.1)	6 (6.9)	5 (5.4)	0.76
Bilateral	175 (94.1)	87 (93.5)	88 (94.6)	

Lower lobe involvement				
Yes	180 (96.8)	88 (94.6)	92 (98.9)	0.09
No	6 (3.2)	5 (5.4)	1 (1.1)	

Additional findings				
Cardiomegaly	85 (45.7)	33 (35.5)	52 (55.9)	0.005
Mosaic attenuation	7 (3.7)	3 (3.2)	4 (4.3)	1
Pleural effusion	35 (18.8)	9 (9.7)	26 (28.0)	0.001
Unilateral	12 (6.5)	3 (3.2)	9 (9.7)	0.07
Bilateral	23 (12.4)	6 (6.5)	17 (18.3)	0.01
Pericardial effusion	13 (7.0)	3 (3.2)	10 (10.8)	0.04
Emphysema	7 (3.8)	3 (3.2)	4 (4.3)	1
Dilated pulmonary trunk	23 (12.4)	5 (5.4)	18 (19.4)	0.004
Pleural thickening	34 (18.3)	3 (3.2)	31 (33.3)	<0.001
Subsegmental atelectasis	64 (34.4)	24 (25.8)	40 (43.0)	0.01

Other morphologies				
Parenchymal band	83 (44.6)	42 (45.2)	41 (44.1)	0.88
Crazy paving	69 (37.1)	29 (31.2)	40 (43.0)	0.09
Reverse halo	14 (7.5)	7 (7.5)	7 (7.5)	1
In-lesion bronchiectasis	11 (5.9)	7 (7.5)	4 (4.3)	0.35

^*∗*^Mean (standard deviation); all other variables reported as N (%). PI = pulmonary involvement; RUL = right upper lobe; RML = right middle lobe; RLL = right lower lobe; LUL = left upper lobe; LLL = left lower lobe; GGO = ground-glass opacity.

**Table 3:** Univariate and multivariate Cox regression.

Variable	Coefficient of variable in the model	Exp(B) (95% CI)	*p* value of variables	*p* value of models
Univariate Cox regression				
PI score ≥ 10	0.60	1.8 [1.09–3.01]	0.02	0.02
PI density score ≥ 2.2	0.57	1.77 [1.10–2.91]	0.03	0.03

Multivariate backward Cox regression				
PI score ≥ 10	0.56	1.75 [1.05–2.9]	0.03	0.01

PI: pulmonary involvement; CI: confidence interval.

## Data Availability

All the data used in this manuscript can be made available upon request to the corresponding author.
